# Epidemiology and Outcomes of Candidemia in a Referral Center in Tehran

**DOI:** 10.22088/cjim.10.1.73

**Published:** 2019

**Authors:** Mohammadreza Salehi, Zahra Ghomi, Reza Mirshahi, Seyed Ali Dehghan Manshadi, Omid Rezahosseini

**Affiliations:** 1 Department of Infectious and Tropical Diseases, Imam Khomeini Hospital Complex, Tehran University of Medical Sciences, Tehran, Iran.; 2 School of Medicine, Tehran University of Medical Sciences, Tehran, Iran.; 3 Eye Research Center, Farabi Hospital, Tehran University of Medical Sciences, Tehran, Iran

**Keywords:** Candidemia, Candida albicans, Mortality, Morbidity

## Abstract

**Background:**

Bloodstream infection with *Candida*, or candidemia, is the most common *Candida* systemic infection. In this study, we investigated the characteristics of patients with candidemia to provide appropriate perspectives on these patients and reduce the associated mortality and morbidity.

**Methods:**

In this cross-sectional study, all patients with at least one positive blood culture of *Candida *spp. were investigated from April 2015 to March 2016 in Imam Khomeini Hospital Complex, Tehran, Iran.

**Results:**

A total of 74 patients (44 men and 30 women), with the mean age of 53.15±17.89 years, were enrolled in this study.* Non-albicans Candida* species was responsible for candidemia in 67.6% (50.74). The mean therapy intervals were 7 and 5.6±1.5 days in patients who died and were discharged, respectively. The differences in frequencies of urinary catheter and mechanical ventilation were statistically significant among patients who died and survived (P<0.001). Among the discharged patients, antifungal therapy was administered to 30.8% (12.39). The mortality rate was 54.3% (19.35) in the medical ward, 5.7% (2.35) in the surgical ward, and 40% (14.35) in the intensive care unit (P=0.041). The treatment was significantly associated with lower mortality than those with no treatment (OR=0.150 [0.023-0.996], P=0.05).

**Conclusion:**

The number of candidemia cases caused by *non-albicans* *Candida* species is continuously increasing in our center. We demonstrated the epidemiologic characteristics of patients with candidemia and the significant effects of timely and appropriate treatment on their outcomes. Further studies are needed to illuminate more aspects of this healthcare problem.

Systemic fungal infections are one of the most common causes of hospital-acquired infections, especially in critically ill patients. Meanwhile, *Candida* comprises a high proportion of these infections (1). *Candida*, as part of normal human mucosal membrane flora, can cause systemic infections in certain conditions, such as mucosal membrane disruption, immunodeficiency, malignancies, renal failure, uncontrolled diabetes, post-surgical procedures, low birth weight or prematurity, and long-term antibiotic use (2-5). Bloodstream infection with *Candida*, or candidemia, is the most common *Candida* systemic infection, which causes serious problems and increases costs in healthcare systems and hospitals (6-8). The recent incidence of candidemia has dramatically increased with the increase in the number of susceptible patients (9).

Although most patients with candidemia are from the intensive care units (ICUs), this infection can also occur in other hospital wards (10) and is known as one of the most common causes of mortality and morbidity in healthcare centers, with a mortality rate of nearly 71% (10-12).

Although *Candida albicans* is the most common cause of candidemia worldwide, cases of infection caused by non-*albicans* species have been increasing recently. Non*-albicans Candida* species were more common in some studies (13, 14), which causes a major dilemma in the treatment of infected patients. These changes are associated with different etiologies. For example, the prophylactic or preemptive use of antifungals such as azoles, inhibit the susceptible species, but cannot affect the resistant species; therefore, its spread cannot be prevented (15, 16). The proliferation of resistant species results in the use of other antifungal agents, such as echinocandines or amphotericin B (17-19). According to the latest version of the Infectious Diseases Society of America (IDSA) guideline for candidiasis treatment, published in 2016, echinocandines (such as caspofungin) were recommended as the first-line treatment for candidemia in adults with and without neutropenia, and fluconazole was recommended as the second line (20). However, currently, some patients do not receive appropriate treatment at the right time. Despite the knowledge about candidemia, many aspects of this infection remain unknown. In fact, physicians face challenges due to epidemiological differences associated with *Candida* species and their antifungal resistance patterns (21). 

Knowing the epidemiological characteristics and outcome-associated factors of candidemia in every healthcare setting will help physicians choose the best treatment approach and reduce the morbidity and mortality. In this study, we investigated the characteristics of patients with candidemia at Imam Khomeini Hospital Complex of Tehran to provide the appropriate perspectives on these patients and reduce their mortality and morbidity.

## Methods

In this cross-sectional study, we evaluated all patients with candidemia from April 2015 to March 2016 in Imam Khomeini Hospital Complex, a referral tertiary center in Tehran, Iran, with several medical, surgical, pediatric, oncology, and ICU wards. Every patient with at least one positive blood culture of *Candida *spp. was included in the study and in patients with several candidemic episodes, only the first episode was included. The data were extracted from patient’s records. 

We collected the demographic data, including age and sex; immunodeficiency conditions; admission ward; time interval between admission and blood sampling; underlying diseases; use of indwelling vascular or urinary catheters, mechanical ventilation, and/or oral intubation; and colonization site for *Candida*. Previous antibacterial therapy was defined as receiving of at least one oral or parenteral antibiotic in a month preceding diagnosis of candidemia (22). Moreover, information on empiric antifungal therapy during the first 48 h after the blood culture, antifungal treatment, time interval between the initiation of therapy and following blood culture, treatment duration, and outcome (mortality vs. discharge) were collected. 

The study was approved by the Ethics Committee of Tehran University of Medical Sciences. Data were analyzed by the IBM-SPSS Statistics for windows Version 24 (IBM Corp., Armonk, NY, USA) using the Chi-squared, Mann-Whitney U-test, t-test, and logistic regression model.

## Results

A total of 74 patients (44 men and 30 women), with the mean age of 53.15±17.89 years, were enrolled in this study. Among the 74 patients, 63 (85.1%) had at least one comorbidity at the time of candidemia diagnosis, 23 (31.1%) had cancer, 21 (28.4%) were immunodeficient, 11 (14.9%) had diabetes, and 11 (14.9%) with renal failure. A total of 28 (37.8%) patients had a history of minor or major surgery in last month; 41 (55.4%) and 45 (60.8%) had vascular and urinary catheters, respectively; and 39 (52.7%) were admitted in the medical wards. Seven (9.5%) patients had positive urinary culture for *Candida* spp. and 1 (1.4%) patient had oral candidiasis.Source of candidiasis was not found in rest of the patients.

*Non-albicans Candida* species were responsible for candidemia in 67.6% (50/74) of the patients. None of our patients received antifungal prophylaxis, but 15 (20.3%) received antifungal treatment within 72 h after the blood culture. Among the 15 treated patients, 8 (53.3) and 7 (46.7) had fluconazole and caspofungin, respectively. The mortality rate was 47.3% (35.74). Table 1 presents the patients’ characteristics, risk factors, admission wards, treatments, and crude mortality rate.

**Table 1:** Comparison of patient’s characteristics, risk factors, admission wards and treatments according to candida species

	**C. albicans** **(n=24) 32.4%** **n(%)**	**C. non-albicans** **(n=50) 67.6%** **n(%)**	**All** **(n=74) 100%** **n(%)**
Male sex, n (%)	14 (58.3)	30 (60)	44 (59.5)
Mean age (SD)	53.2 (18.8)	53.1 (17.5)	53.1 (17.8)
**Risk Factors**			
Vascular Catheter	13 (54.2)	28 (56)	41 (55.4)
Urinary Catheter	14 (58.3)	31 (62)	45 (60.8)
Mechanical Ventilation	12 (50)	21 (42)	33 (44.6)
Surgury Hx	12 (50)	16 (32)	28 (37.8)
DM	1 (4.2)	10 (20)	11 (14.9)
Immunodeficiency	6 (25)	15 (30)	21 (28.4)
Renal failure	4 (16.7)	7 (14)	11 (14.9)
Hepatic Failure	3 (12.5)	2 (4)	5 (6.8)
Bedridden	1 (4.2)	3 (6)	4 (5.4)
CHF/IHD	3 (12.5)	3 (6)	6 (8.1)
Cancer	9 (37.5)	14 (28)	23 (31.1)
Neurologic disorder	3 (12.5)	7 (14)	10 (13.5)
**Admission Ward**			
Medical	13 (54.2)	26 (52)	39 (52.7)
Surgical	4 (16.7)	5 (10)	9 (12.2)
Hematology/Oncology	0 (0)	4 (8)	4 (5.4)
ICU	7 (29.2)	15 (30)	22 (29.7)
**Empirical Treatment**			
Fluconazole	4 (80)	4 (40)	8 (53.3)
Caspofungin	1 (20)	6 (60)	7 (46.7)
Mortality	12 (50)	23 (46)	35 (47.3)

The median age of patients was 56 years. Hence, we divided the patients into two age groups (<55 and >55 years) and analyzed their characteristics accordingly. The frequency of urinary catheter use was 47.2% (17.36) and 73.7% (28.38) in patients aged <55 and >55 years, respectively (P=0.02). Among the patients with cancer, 16.7% (6.36) and 44.7% (17.38) were aged <55 and >55 years, respectively (P=0.009). 

Table 2 presents the clinical characteristics of patients aged <55 and >55 years.

The mean therapy interval was 7 days in patients who died and 5.6±1.5 days in patients who were discharged. The differences in the frequencies of urinary catheter and mechanical ventilation use were statistically significant among patients who died and were discharged (P<0.001). Among the patients who died, 11.4% (4.35) were bedridden (P=0.03). Among the discharged patients, antifungal therapy was administered to 30.8% (12.39), and 53.3% (8.12) of them were immunodeficient (P=0.016). The mortality rate according to the admission record was 54.3% (19.35) in the medical ward, 5.7% (2.35) in the surgical ward, and 40% (14.35) in the ICU (P=0.041). There was no mortality recorded in the hematology/oncology ward. Table 3 compares the characteristics of patients who died and were discharged.

The logistic regression model analyzes the relationship between the use of urinary catheter and mechanical ventilation, bedridden condition, admission ward, and treatment. 

Only the treatment was significantly associated with lower mortality when compared with no treatment (OR=0.150 [0.023-0.996], P=0.05).

**Table 2:** Comparison of the characteristics of the dead and discharged patients

	**Dead** **n (%) N=35**	**Discharged ** **n (%) N=39**	**p-value** *
Male sex, n (%)	12 (65.7)	21 (53.8)	0.423
Mean age ± SD	55.8±20.5	50.7±15.0	0.223
Mean Therapy Duration ± SD	6.0± 1.0	12.8 ± 6.5	0.102
Mean Therapy Interval ± SD	7.0 ± 0.0	5.6 ± 1.5	0.008
**Risk Factors**			
Vascular Catheter	21 (60)	20 (51.3)	0.451
Urinary Catheter	29 (82.9)	16 (41)	<0.001
Mechanical Ventilation	24 (72.7)	9 (23.1)	<0.001
Surgury Hx	10 (28.6)	18 (46.2)	0.119
DM	3 (8.6)	8 (20.5)	0.149
Immunodeficiency	6 (17.1)	15 (38.5)	0.420
Renal failure	6 (17.1)	5 (12.8)	0.602
Hepatic Failure	3 (8.6)	2 (5.1)	0.556
Bedridden	4 (11.4)	0 (0.0)	0.030
CHF/IHD	3 (8.6)	3 (7.7)	0.890
Cancer	10 (28.6)	13 (33.3)	0.659
Neurologic disorder	5 (14.3)	5 (12.8)	0.854
**Admission Ward**			
Medical	19 (54.3)	20 (51.3)	**0.041**
Surgical	2 (5.7)	7 (17.9)	
Hematology/Oncology	0 (0.0)	4 (10.3)	
ICU	14 (40)	8 (20.5)	
**Candida Species**			
C. Albicans	12 (34.3)	12 (30.8)	0.747
**Empirical Treatment**			
Yes	3 (8.6)	12 (30.8)	0.018
Fluconazole	1 (33.7)	7 (58.3)	0.438
Caspofungin	2 (66.7)	5 (41.7)	

* p<0.5 is statistically significant

**Table 3:** Comparison of the clinical characteristics of patients under and over 55 years old

	**Under 55 n (%) N=36**	**Over 55 n (%) N=38**	**p-value** *
Male sex, n (%)	19 (25)	25 (65.8)	0.255
Mean Rank of Therapy Duration (Day)	8.28	7.58	0.766^
Mean Therapy Interval ± SD (Day)	5.7±1.4	6.2±1.6	0.535
**Risk Factors**			
Vascular Catheter	19 (52.8)	22 (57.9)	0.658
Urinary Catheter	17 (47.2)	28 (73.7)	0.020
Mechanical Ventilation	14 (38.9)	19 (50)	0.337
Surgery History	13 (36.1)	15 (39.5)	0.766
Diabetes	3 (8.3)	8 (21.1)	0.124
Immunodeficiency	14 (38.9)	7 (18.4)	0.051
Renal failure	3 (8.3)	8 (21.1)	0.124
Hepatic Failure	1 (2.8)	4 (10.5)	0.184
Bedridden	3 (8.3)	1 (2.6)	0.278
CHF/IHD	1 (2.8)	5 (13.2)	0.102
Cancer	6 (16.7)	17 (44.7)	0.009
Neurologic disorder	3 (8.3)	7 (18.4)	0.205
**Admission Ward**			
Medical	23 (63.9)	16 (42.1)	0.051
Surgical	6 (16.7)	3 (7.9)	
Hematology/Oncology	1 (2.8)	3 (7.9)	
ICU	6 (16.7)	16 (42.1)	
**Candida Species**			
C. Albicans	12(33.3)	12 (31.6)	0.872
**Empirical Treatment**			
Yes	9 (25)	6 (15.8)	0.325
Fluconazole	4 (11.1)	4 (10.5)	0.437
Caspofungin	5 (13.9)	2 (5.3)	
**Mortality**			
Dead	13 (36.1)	22 (57.9)	0.061

* p<0.5 is statistically significant

^ Mann-Whitney U-test was applied

## Discussion

Although *Candida albicans* is still known as the dominant species responsible for candidemia, *non-Albicans* species have shown an increasing trend recently. *Non-albicans Candida* species were responsible for candidemia in 67.6% of our patients, which has been consistent with the findings in recent studies (23, 24). However, some specific predisposing factors of each *non-albicans Candida* species infection (e.g., indwelling urinary catheter for *Candida parapsilosis* and malignancy for *Candida glabrata* or *Candida* *krusei*) were inconsistent with our results (25-27). The confounding effects of our laboratory facilities, lacking the ability to distinguish various *non-albicans Candida* species, should also be considered.

Candidemia is a hospital-acquired infection with high mortality rate (28). The mortality rate in our study was 47.3%, which is similar to that of the previous studies (28). The minimal differences in mortality rate worldwide can be explained by the differences in treatment strategies or medications, antifungal drug resistance, and epidemiologic characteristics of the target population. 

The mortality rate in patients with candidemia varies among the different wards. Several evidence supports the fact that patients admitted in the ICU and medical ward have higher mortality rates than those in other wards (28). Marriot et al. reported a mortality rate of 56% among ICU patients with candidemia (29), which is almost similar with our data showing that the highest mortality rate was observed among patients in the medical ward (54.3%), followed by the ICU (40%), and the lowest mortality rate was observed in patients admitted in the hematology/oncology ward. This difference may be due to the severity of the underlying disease, comorbidities, and poor general condition of patients admitted in medical wards and ICUs. However, the severity scores of our patients were not calculated.

Prolonged antibacterial therapy is a risk factor for candidemia (2-4), although 68.9% of our patients had a history of previous antibacterial therapy, but we could not find any significant association between the type of candidemia or age group with this risk factor. Older patients had higher frequency of urinary catheter use and cancer in our study. Urinary catheters are a source of fungal colonization and infection (30), and cancers are favorable conditions for these infections. Older patients had an insignificant but higher mortality rate than the younger patients. The outcome of candidemia greatly depends on the administration of proper treatment at the right time. In fact, lack of proper treatment is known as an important modifiable risk factor for mortality in these patients (31). A study on 753 patients with candidemia in the United States reported a significant better outcome in patients who received timely appropriate treatments (32). Our findings were consistent with the previous studies that observed low mortality among patients who received appropriate treatment. The low mortality rate among immunodeficient patients in this study can be explained by their high antifungal treatment.

We only treated 20.3% of patients with candidemia, which was approximately 50% than those in the previous studies (29, 31). This along with the fact that none of our patients took prophylaxis for fungal infections indicates that fungal infections including candidemia are vastly underestimated and disregarded. 

Treatment using echinocandins such as caspofungin or micafungin was associated with low mortality in previous studies (32, 33). Nevertheless, studies comparing the efficacy of ecinocandins and azole are limited (34). One of the few articles in this field compared the efficacy of anidulafungin and fluconazole on 345 patients with candidemia (34). The result showed greater success rate with anidulafungin (75.6% successful treatment with anidulafungin vs. 60.2% with fluconazole) (34). Nonetheless, our study failed to detect any difference in the outcome among patients who received different treatment medications (fluconazole vs. caspofungin). This is probably due to a small population of treated patients (20.3%); therefore, further studies with a larger target population should be conducted. This study also has several limitations. These include lack of control group, which prevented us from evaluating the risk factors and attributed mortality rate for candidemia; small target population (especially for treated patients), which interfered with the statistical analysis; non-differentiation of fungal subspecies; and inefficient data collecting systems in our center, which caused some missing data. 

In summary, *non-albicans Candida* species seem to be an increasing important cause of candidemia. We demonstrated the epidemiologic characteristics of patients with candidemia and the significant effects of timely appropriate treatment on their outcomes. Further studies are needed to illuminate more aspects of this healthcare problem and to determine effective treatment strategies for better management of patients with candidemia. 
